# Association of Enhanced HIV-1 Neutralization by a Single Y681H Substitution in gp41 with Increased gp120-CD4 Interaction and Macrophage Infectivity

**DOI:** 10.1371/journal.pone.0037157

**Published:** 2012-05-14

**Authors:** Rajesh Ringe, Jayanta Bhattacharya

**Affiliations:** Department of Molecular Virology, National AIDS Research Institute, Indian Council of Medical Research, Bhosari, Pune, India; University of Massachusetts Medical Center, United States of America

## Abstract

HIV-1 variants that show unusual sensitivity to autologous antibodies due to presence of critical neutralization signatures would likely contribute towards rational envelope based HIV-1 vaccine design. In the present study, we found that presence of a naturally occurring H681 in gp41 membrane proximal external region (MPER) of a clade C envelope (Env) obtained from a recently infected Indian patient conferred increased sensitivity to autologous and heterologous plasma antibodies. Furthermore, Env-pseudotyped viruses expressing H681 showed increased sensitivity to soluble CD4, b12 and 4E10 monoclonal antibodies both in related and unrelated Envs and was corroborated with increased Env susceptibility and binding to cellular CD4 as well as with prolonged exposure of MPER epitopes. The increased gp120-CD4 interaction was further associated with relative exposure of CD4-induced epitopes and macrophage infectivity. In summary, our data indicate that Y681H substitution exposes neutralizing epitopes in CD4bs and MPER towards comprehensive interference in HIV-1 entry.

## Introduction

It is widely believed that a successful and protective vaccine to Human Immunodeficiency virus Type 1 (HIV-1) will lie in its ability to induce broadly neutralizing antibody (NAb) response [1]. Though infection with HIV-1 results in antibody response to most viral proteins, but only antibodies to the surface envelope (Env) are capable of mediating virus neutralization and restrict entry [2], [3]. Thus, identification of signatures in Env that optimally exposes epitopes that are targets of broadly neutralizing antibodies are highly sought [4].The HIV-1 Env is heavily glycosylated and composed of three identical surface gp120 monomers, each non-covalently associated with a transmembrane gp41 molecule [2], [5], [6]. These trimeric Env spikes are responsible for interacting with cell surface CD4 and a coreceptor to initiate viral entry. It is believed that the binding of NAbs to native gp120 trimers is necessary for efficient neutralization [7]. However, the inherent instability of the HIV-1 envelope (Env) spikes has presented challenges to the development of native recombinant trimers [8]. This suggests that the ability of neutralizing antibodies (NAbs) in abrogating HIV-1 entry is dependent on conformational stability of gp120 required for optimum CD4 binding and/or interfering fusion of viral and cellular membrane mediated by gp41. Although antibodies targeting gp41 and gp120 variable regions is detected as early as three weeks after infection [9], [10], detectable NAbs against the viral Env are usually generated only after several months of infection [11]. Importantly, the early NAb response is subsided by successive escape of virus from autologous antibodies [12], [13], [14], [15], [16], [17] for continued high-level of virus replication towards progressive destruction of CD4^+^ T cells, development of acquired immunodeficiency syndrome (AIDS).

Env has a complex structure and upon gp120 engagement with CD4 receptor it undergoes substantial rearrangements in its conformation that facilitates virus fusion with host cell [18], [19], [20]. The precise mechanism of how Env in the CD4-bound configuration undergoes conformational changes is not understood clearly, however it is generally believed that the conformational switch of Env from the unliganded to the CD4-bound state is probably modulated by topological layers present in the inner domain of gp120 that are believed to be flexible in nature [21]. Finzi *et al* [21] recently demonstrated that, as CD4 comes in contact with the Env outer domain, the topological layers 1 and 2 interact in such a way that this resulted in strengthening gp120-CD4 interaction. Although HIV-1 Env is the principal target for NAbs and neutralizing epitopes are present in both Env subunits [22], several targets in Env vulnerable to antibody neutralization are not present on the trimeric form of virion-associated Env, such as V3 loop, CD4-induced epitopes (CD4i) and CD4 binding site (CD4bs) [23], [24], [25]. Despite this variations in presentation of neutralizing epitopes, patients have been shown to develop robust NAb titers to their autologous antibodies [26], [27], [28], [29], [30]. It is of interest that due to fitness constraints some viruses do not undergo rapid evolution and thus become significantly less resistant to neutralization [13], [31], [32] and as such certain epitopes on Env present in these viruses remain vulnerable to neutralizing antibodies. Hence, epitopes in Env that remains unchanged irrespective of virus evolution under immense humoral pressure and are targets of broad and potent neutralizing antibodies such as the newly discovered monoclonal antibodies (MAbs) that targets CD4bs and quaternary epitopes (QNE) [32], [33], [34] are of particular interest and could potentially contribute towards a successful vaccine development. Some of these epitopes are well studied, while others like those present on variable loops for newly discovered broadly neutralizing monoclonal antibodies (bNAbs such as PG9 and PG16 [34], [35], [36] are being studied in depth [11], [37], [38]. In addition to sites on gp120, the membrane proximal external region (MPER) of gp41 that is formed by a linear sequence of 30 amino acids is vulnerable to NAbs [11]. MPER in gp41 is the target of three known neutralizing monoclonal antibodies (MAbs); 2F5, 4E10, and Z13e (34–36), although few others have been described recently [39], [40]. Antibodies that bind to the MPER are believed to block viral fusion to the host cell membrane, and thus abrogate viral entry (59). Though these MAbs, especially 4E10 targeting MPER have shown remarkable breadth, they appear to be uncommon during natural infection (40, 60–62). Interestingly, viruses that escape the autologous serum NAb response has been shown to generally remain sensitive to neutralization by b12, 2G12, and the MPER-directed MAbs suggesting that the initial NAb response is least likely directed to these more conserved epitopes on gp120 [11]. The MAbs 2F5 and 4E10 have been shown to be unusually hydrophobic and auto reactive [41], [42] due to which these antibodies possibly face problems in gaining easy access to conserved regions of HIV-1 during natural infection. Very recently, it was shown that residues in gp41 modulated neutralization sensitivity of HIV-1 Env [43], [44], suggesting that gp41 with relatively highly conserved sequences than that of gp120 may provide better targets for comprehensive virus neutralization. Recently, we showed that a single amino acid substitution (I424M) in C4 region of gp120 exposed vulnerable neutralizing epitopes in CD4bs and V3 loop in a clade C Env [45] obtained from an Indian patient (IVC-4) with recent infection [46]. In the present study, we examined the basis of enhanced sensitivity of one *env* clone to autologous plasma antibodies compared to the contemporaneous env clones amplified simultaneously at the base line visit from the same patient (IVC-4) [46]. On further investigation, we found that a single Y681H substitution in the gp41 MPER conferred increased sensitivity of Env-pseudotyped viruses to antibodies targeting CD4bs and MPER and was associated with increased macrophage infectivity.

## Results

### A single Y681H substitution in gp41 conferred enhanced Env sensitivity to autologous and heterologous plasma antibodies

**Figure 1:**
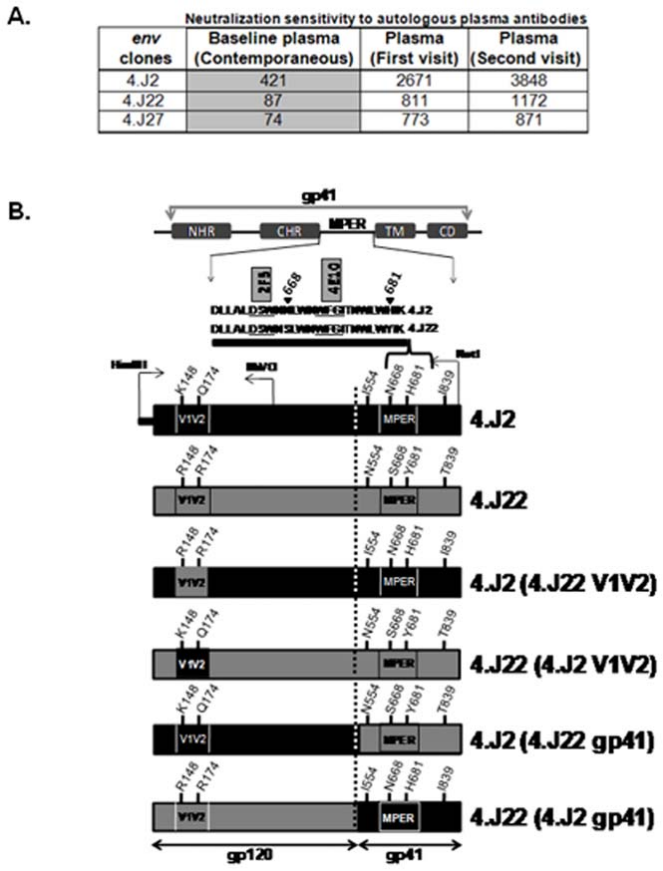
Construction of chimeric envelopes. Chimeric Envs were constructed by swapping of gp120 and gp41 between sensitive (4.J2) and resistant (4.J22) patient Envs using NotI, BbvCI and HindIII restriction enzymes. The ID50 values of pseudotyped viruses carrying 4.J2, 4.J22 and 4.J27 Envs to autologous plasma reported earlier [46] were shown on top; highlighted column represents ID50 of Env-pseudotyped viruses to contemporaneous plasma at the base line.

**Figure 2:**
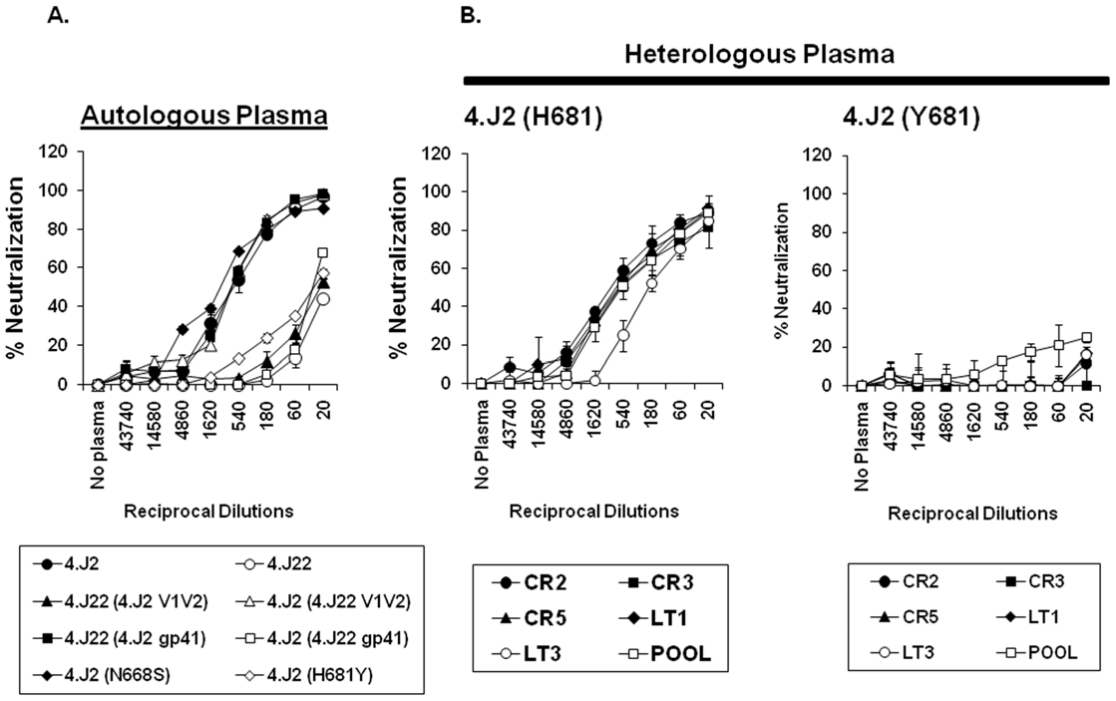
Neutralization of patient Envs by autologous and heterologous plasma antibodies. A. Neutralization curves of Env-pseudotyped viruses carrying chimeric Envs as well as with point substitutions at positions 668 and 681 were tested against autologous plasma antibodies. Percent neutralization at various dilutions of plasma is plotted and compared with virus control (VC) where growth medium was used instead of plasma. Note that the gp41 has modulated the neutralization sensitivity to autologous plasma and specifically H681Y substitution in 4.J2 Env was found to associate with reduced neutralization sensitivity to autologous plasma. B. 4.J2 Env carrying H681 was found to confer enhanced sensitivity to HIV-1^+^ heterologous plasma samples (CR2, CR3, CR5, LT1, LT3) while Y681 rendered significant reduction of 4.J2 Env sensitivity to same heterologous plasma samples. Plasma pool represents the mixture of above five plasma specimens. The neutralization assays were done in duplicate and in at least three independent experiments. In all cases, VC stands for virus control where plasma was not added and thus considered as 0% neutralization.

We previously described [46] two clade C Env clones 4.J2 and 4.J22 obtained from a recently infected Indian patient at the baseline (at the same time point) which differed in their sensitivities to autologous plasma antibodies (ID_50_ of 4.J2 = 1∶540 against ID_50_ of 4.J22<100) (Figure 1A). The Env clones obtained were within one year of infection as determined by detuned ELISA as described earlier [46]. Sequence comparison between these two Envs revealed differences of altogether six residues in gp160, two in V1V2 domain (at positions 148 and 174 respectively) of gp120 and four in gp41 (Figure 1B). Out of the four residues that differed between these two Envs in gp41 domain, two were in the MPER region at positions 668 and 681, while the two others were at positions 551 and 839. We first investigated domains in Env that modulated sensitivity to contemporaneous autologous plasma by constructing Env chimeras between 4.J2 and 4.J22. Pseudotyped viruses carrying chimeric Env constructs were tested for their neutralization sensitivity to autologous contemporaneous plasma antibodies. As shown in Figure 2, as opposed to the wild type, 4.J2 containing gp41 grafted from 4.J22 conferred resistance while 4.J22 containing gp41 from 4.J2 became sensitive to autologous antibodies, indicating that gp41 in these Envs conferred altered sensitivities to autologous plasma antibodies in this patient. Since residues at positions 668 and 681 in these Envs were within MPER, a region of effective target of neutralizing antibodies, and in very close proximities to motifs that are targets of 2F5 and 4E10, we substituted N668S and H681Y (668S and 681Y were present in resistant Env 4.J22) in the sensitive 4.J2 Env and tested against autologous plasma antibodies. As shown in Figure 2, we found that while 4.J2 (N668S) retained similar sensitivity to autologous plasma as 4.J2, 4.J2 (H681Y) became resistant, suggesting that Y681 was responsible for neutralization resistance of 4.J22 Env clone in this patient. Interestingly, when tested against heterologous plasma antibodies obtained from five chronically infected patients, 4.J2 (containing H681) showed increased sensitivity while 4.J2 (H681Y) was found to be resistant (Figure 2). Our data indicated that H681 enhanced neutralization possibly by altering Env conformations.

### Y681H altered the exposure of neutralizing epitopes in MPER

**Figure 3:**
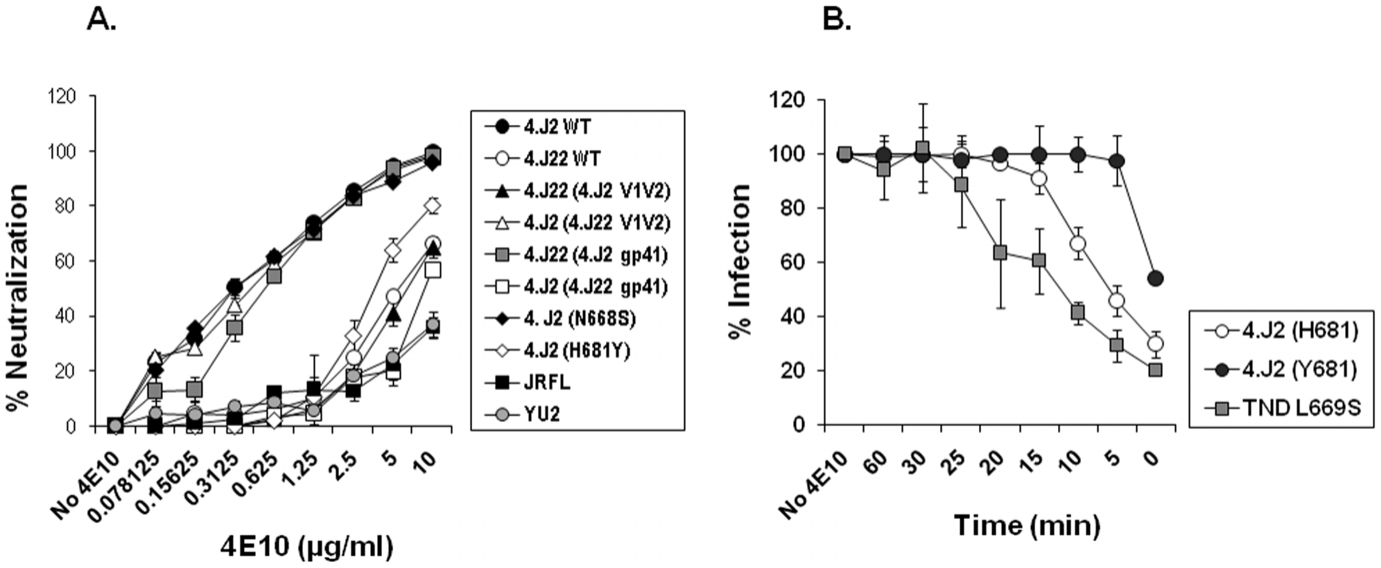
Effect of Y681H substitution on exposure of neutralizing epitopes in gp41 MPER. A. Neutralization sensitivity of Env-pseudotyped viruses expressing Y681 and H681 were tested against 4E10 MAb in TZM-bl cells. Percent neutralization on Y-axis against serial dilutions of 4E10 indicated on X-axis shows H681 conferring increased Env sensitivity to 4E10 MAb (and also T20; see Figure S1 and Table 1). The neutralization assays were done in duplicate in more than three independent experiments. VC stands for virus control where antibody was not added. B. Time course of 4E10 MAb neutralization of Envs. 4E10 was added in warm condition (37°C) at different time points in TZM-bl cells which were pre-adsorbed with Env pseudotyped virus in cold condition. The percent infection is plotted on Y-axis against various time points of 4E10 addition given on X-axis. No 4E10 indicates infection of TZM-bl cells in absence of 4E10 MAb and hence serves as virus control. Note that 4E10 could neutralize 4.J2 (H681) by 50% up to 6 min as opposed to 1 min for 4.J2 (Y681). TND S669 that was earlier reported [44] for enhanced exposure of neutralizing epitopes in MPER was used as positive control.

Since H681 was located in MPER, to further examine if resistance to autologous neutralization conferred by H681Y was due to its effect on MPER, we tested the sensitivity of pseudoviruses carrying 4.J2 (H681) and 4.J2 (Y681) Envs to 4E10 MAb. We did not test the sensitivities of these Envs to 2F5 as they were found to be resistant to 2F5 because of lack of minimum DKW motif for 2F5 recognition [46]. As shown in Figure 3A, we found that while 4.J2 (N668S) did not alter 4E10 sensitivity, H681Y conferred 4E10 resistance by greater than 16-folds. Enhancement of Env sensitivity to 2F5 was also found with unrelated Envs with Y681H substitutions (Table 1), although it was not as dramatic as 4E10. Our data indicated that presence of H681 modulated Env conformation at MPER and increased neutralization by antibodies targeting this region.

**Table 1:** Neutralization properties of Envs carrying Y681 and H681 in different genetic backgrounds.

IC50 (µg/ml)							
Envs	Clade	b12	sCD4	4E10	2F5	T20	Pooled Plasma (Reciprocal Dilutions)
**4.J2**	C	>25	0.625	0.28	No motif †	0.0054	1011
**4.J2 (H681Y)**	C	>25	>25 (>40*)	4.2 (15)	No motif	0.008	102 (9.9*)
**11-3.J3**	C	>25	>25	5.64	>25	0.2	443
**11-3.J3 (Y681H)**	C	>25	4.49 (>6*)	1.76 (3.2*)	12.5 (>2*)	0.015 (13.33*)	8266 (18.65*)
**2-3.J7**	C	2.2	>25	6.0	No motif†	0.165	161
**2-3.J7 (Y681H)**	C	0.31 (8.8*)	4.5 (>6*)	1.3 (4.6*)	No motif	0.0353 (4.67*)	354 (2.2*)
**YU2**	B	3.5	1.1	25	>25	0.9	134
**YU2 (Y681H)**	B	0.25 (14*)	<0.078 (>15*)	0.83 (30.1*)	1.86 (>14*)	0.09 (10*)	418 (3.12*)
**Q259ENVd2.17**	A	>25	>25	>25	>25	0.50	82
**Q259ENVd2.17 (Y681H)**	A	6.74 (>4*)	6.85 (>4*)	1.76 (>14*)	>25 (none)	0.02 (25*)	128 (1.56*)
						0.0078	105
**CRF-02_AG-235**	A/G	>25	>25	4.1	>25	0.0039 (2*)	930 (8.85*)
**CRF-02_AG-235 (Y681H)**	A/G	0.83 (>30*)	0.2 (>125*)	0.12 (34.16*)	>25 (none)		
**TND_L669**				8.054		NOT DONE	120
**TND_L669S**	B	2.06	10	0.031 (259.8*)	3.915	NOT DONE	355
**Q461.e2**	B	0.53 (3.88*)	1.25 (8*)	>25	0.014 (279.57*)	NOT DONE	60
**Q461.e2 (TA)**	A	>25	>25	15 (1.6*)	>25	NOT DONE	125
**Q461.e2 (IV)**	A	>25 (NONE)	1 (>25*)	1.0 (>25*)	10.8 (>2.31*)	NOT DONE	180
	A	>25 (NONE)	5.0 (>5*)		0.92 (>27.1*)		

* Represents fold-increase in sensitivity of Env-pseudotyped viruses expressing H681 compared to those expressing Y681.

† The minimum DKW motif necessary for 2F5 recognition is absent.

The overall differences in sensitivity of Envs expressing H681 with that of Y681 to sCD4 and 4E10 MAb were significant (P = 0.0002 and P = 0.008).

TND-L669 and TND_L669S [44] as well as Q461.e2, Q461.e2 (TA) and Q461.e2 (IV) [43] that were earlier reported to enhance sensitivity of HIV-1 to neutralizing antibodies were tested in parallel. Fold differences in neutralization sensitivities are given in parentheses.

Since 4E10 MAb targets the structural intermediate formed during the fusion process we further explored if increased sensitivity of Envs to antibodies targeting MPER in presence of H681 was due to alteration in fusion kinetics. Hence, we tested the sensitivities of Envs with both Y681 and H681 to T20, a classical fusion inhibitor that inhibits 6-helix bundle formation and prevent fusion, at different doses. As shown in Table 1, Y681H indeed exhibited enhanced inhibition of Envs by T20 in a dose-dependent manner (Figure S1). Statistical analysis of paired IC50 values (H681 versus Y681) by one tailed t-test showed significant increase in sensitivity of H681 Env variants to T20 (P = 0.04), suggesting that presence of H681 indeed interfered with fusion efficiency and likely resulted in altered exposure of neutralizing epitopes in MPER. Since differences in T20 sensitivities by Envs indicated altered kinetics of structural transition of gp41 during fusion due to Y681H substitution, we further examined if this phenomenon leads to differences in exposure of MPER. To test this we carried out virus–cell post-attachment neutralization assay using 4E10 MAb as described recently [44] and measured the infectivity of pseudoviruses 4.J2 (H681) and 4.J2 (Y681) as a function of time of addition of 4E10 MAb. Briefly, Env-pseudotyped viruses were adsorbed in TZM-bl cells on ice for 2 hours. Subsequently, unbound viruses were removed by successive washing with cold medium. To examine how 4E10 MAb arrests virus fusion with TZM-bl cells, 4E10 MAb (10 µg/ml) was added to TZM-bl cells pre-adsorbed with Env-pseudotyped viruses at different time intervals. As shown in Figure 3B, we found that while 4E10 neutralization of 4.J2 Env (containing H681) was prolonged and was effective up to 15 minutes post-attachment, presence of Y681 rendered 4.J2 (H681Y) with rapid fusion (within 5 minutes). Sensitivity to T20 and time course of 4E10 neutralization, taken together, suggested that slower entry of virus into the cell possibly due to altered fusion kinetics upon Y681H substitution leading to prolonged exposure of MPER epitopes.

### Effect of Y681H substitution on CD4 binding sites in gp120

**Figure 4:**
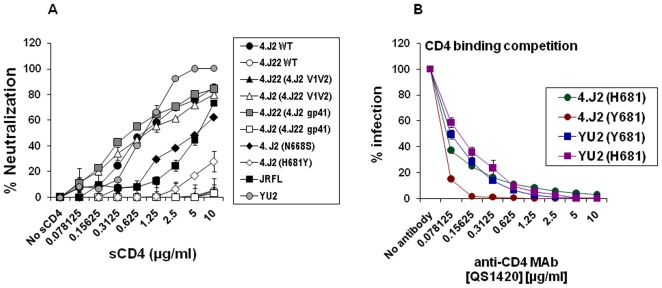
Effect of presence of H681 on gp120-CD4 interaction. A. Neutralization sensitivity of Env-pseudotyped viruses to sCD4 was carried out in TZM-bl cells. Percent neutralization was measured by scoring RLU as indicated on Y-axis at various concentrations of sCD4 plotted on X-axis. B. Competition between Env-pseudotyped viruses (carrying H681 and Y681) and anti-CD4 MAb QS1420 for binding to cellular CD4. Env pseudotyped virus was expected to compete with QS1420 to bind with the cellular CD4 on TZM-bl cells for productive infection. Percent infection of Env pseudotyped virus on Y-axis in presence of various concentration of QS1420 on X-axis was recorded in comparison with infectivity of virus in absence of QS1420 which was considered as 100%. Percent infectivity was assessed by measuring RLU in a luminometer. The result in the graph is representative of a single experiment performed in duplicate. The assay was performed at three independent time points using an independent virus preparation.

**Figure 5:**
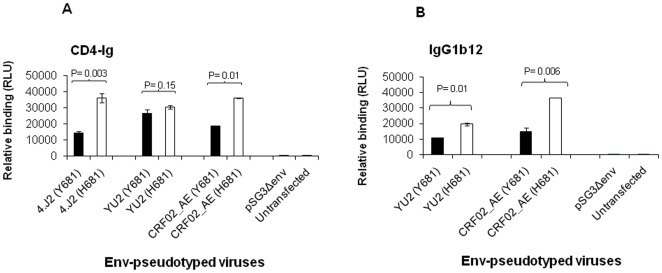
Effect of Y681H on relative binding of gp120 with CD4. Cell-based enzyme-linked Immunosorbant assay (CELISA) was carried out with indicated Envs. The relative binding of (A) CD4-Ig and (B) IgG1b12 to Env trimers expressed on 293T cells was measured as relative luminescence units (RLU) on Y-axis. pSG3Δenv and untransfected 293T cells were taken as negative control.

Neutralization of wild type Envs 4.J2 (containing N668, H681) and 4.J22 (containing S668, Y681) with soluble CD4 showed that 4.J2 was sensitive while 4.J22 being completely resistant at 25 µg/ml. Next we wanted to know what domain has modulated this sensitivity to sCD4. We assessed the neutralization sensitivity of chimeric clones made between 4.J2 and 4.J22, mutant clones of 4.J2 where we substituted H681Y and N668S, and wild type Envs. As shown in Figure 4A, 4.J2 (H681) conferred significantly higher sensitivity to sCD4 over 4.J2 (Y681) by >40-folds, N668S moderately decreased the sensitivity to sCD4. All the Envs which contained H681 showed higher sensitivity to sCD4 in the panel of Envs while those containing Y681 were completely resistant at 10 µg/ml. This data shows that N668 and H681 act in cooperation to enhance the sensitivity of Env to sCD4, although H681 exerts more influence than N668 for sensitivity. The results were quite intriguing as the same H681 which modulated the neutralization sensitivity to 4E10 was found to alter sCD4 sensitivity-the phenotype which is supposedly more relevant to changes in gp120. The data imply that the change in MPER does not only exert its effect at local site in gp41 subunit but also traverse through transmembrane subunit to surface protein gp120 to alter the conformation at CD4 binding site as supported by CD4-Ig and IgG1b12 binding with Env trimers (Figure 5).

To elucidate the cause of enhanced neutralization of H681 variants by sCD4 we carried out Env-anti-CD4 antibody competition assay with TZM-bl cells that express CD4 and CCR5. This assay aimed to measure the binding affinity of Envs (with H681 or Y681) with CD4 expressed on the TZM-bl cells. In this assay, equal quantity of virus was resuspended in various dilutions of anti-CD4 antibody and added on the TZM-bl cells in 96 well tissue culture plate and the infectivity was measured after 48 hours of incubation by measuring luciferase activity. The data showed that as the concentration of anti-CD4 antibody (QS1420) reduced the infectivity of 4.J2 (H681) increased rapidly as compared to 4.J2 (Y681). As shown in Figure 4B, the rate of increase in infectivity of 4.J2 (H681) was found to be ∼5 times higher than 4.J2 (Y681). Similar results were observed with Y681 and H681 versions of YU2 although the difference in affinity of these two was subtle. The subtle increase in affinity of YU2 (H681) might be because intrinsic high sensitivity of YU2 wild type to sCD4 and IgG1b12 and though difference in neutralization sensitivity of H681 and Y681 is apparent the difference in affinity is marginal.

#### Effect of Y681H substitution on interaction of IgG1b12 and CD4-Ig with Env trimers

Next we examined whether there was any association between enhanced neutralization sensitivity due to presence of H681 with increased binding of CD4-Ig and IgG1b12 MAb to Env trimers expressed on the cell surface in cell-based ELISA (CELISA) as described recently [47]. Thus, we examined H681 and Y681 versions in 4.J2, YU2 and CRF02-AG235 Envs that showed enhanced sensitivity to sCD4 for their degree of relative binding to Env trimers expressed on 293T cells. The 4.J2 Envs were not tested against IgG1b12 as they were known to be resistant to this MAb [46]. For both CD4-Ig and IgG1b12, all the Envs expressing H681 showed (Figure 5) significant increase (P<0.05) in their binding relative to those expressing Y681; although YU2 (H681) showed marginal increase in CD4-Ig binding compared to that of Y681, it was not significant (P = 0.15). Although the direct evidence of conformational change is lacking, mounting data from the binding and neutralization assays with reagents that interfered the Env: CD4 interaction suggested that Y681H substitution indirectly altered the Env conformation at CD4 binding site and favors the interaction of Env with these reagents.

### Y681H substitution enhances infectivity in low CD4 expressing cells

**Figure 6:**
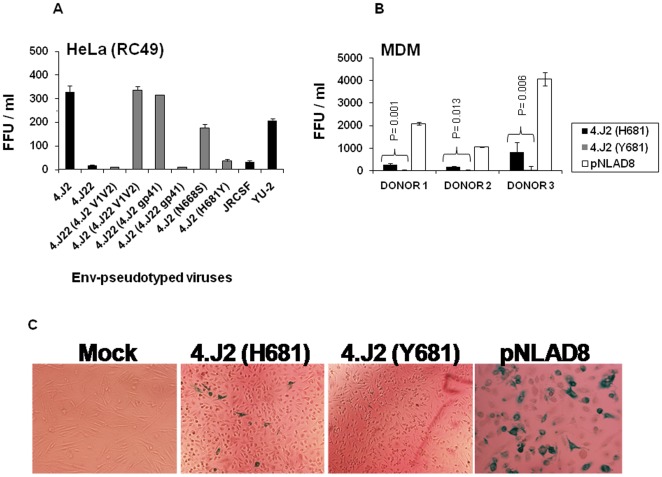
Infectivity of Env-pseudotyped viruses in cells expressing low CD4. Infectivity of Env-pseudotyped viruses was tested in (A) HeLa cells expressing low CD4 (RC49) [48]and (B) monocyte-derived macrophages (MDM). In both cases, effector cells were infected with Env-pseudotyped viruses with equal infectious units and the infectivity was assessed by immunostaining intracellular p24 and expressed as focus forming units (FFU) on Y-axis in a β-galactosidase assay as described earlier [46], [51]. (C). The β-galactosidase positive cells (in blue) representing MDM (from one donor) expressing intracellular p24 were scored as focus forming units (FFU) to assess the relative infectivity. For MDM assays, macrophage tropic pNLAD8 was used as positive. In HeLa (RC49) cells, YU2 and JR-CSF were used as controls.

Since Envs expressing H681 showed enhanced sensitivity to sCD4 (Figure 4) and IgG1b12 (Table 1) and showed relatively increased binding with CD4-Ig and b12 over Envs expressing Y681, we hypothesized that H681 possibly modulated Env conformation at CD4 binding site in gp120. The enhanced interaction of Env trimers with CD4-Ig and IgG1b12 implied that Y681H substitution likely induced exposure of CD4bs enabling Envs towards low CD4 dependence for productive entry into the target cells. Thus, to test this, we examined the effect of N668S and H681Y on CD4 dependence of these Envs by infecting Env-pseudotyped viruses carrying both H681 and Y681 in HeLa cells expressing low CD4 but high CCR5 (Clone RC49) [48]. RC49 cells express high amount of CCR5 coreceptor but limiting amount of CD4 and virus infectivity in these cells is a measure of CD4 dependence of Env for entry. As expected, we found that 4.J2 (H681) showed greater infectivity in RC49 cells (Figure 6A) than 4.J2 (Y681) indicating that H681 modulated Env towards greater CD4bs exposure and possibly enhanced binding with CD4. In comparison to H681Y, N668S substitution in 4.J2 Env showed only modest reduction in infectivity of RC49 cells, suggesting H681 predominantly affected CD4bs in Env. Finally, we examined the effect of increased exposure of CD4bs due to presence of H681 in Env-pseudotyped viruses on their degree of infectivity in monocyte-derived macrophages (MDM) (obtained from three different donors) that naturally express low amounts of cell surface CD4 [49], [50]. Thus, MDMs were infected with equal TZM-bl infectious units of 4.J2 (H681) and 4.J2 (Y681) and infectivity measured by immunostaining of p24 positive cells as described before [51]. As shown in Figure 6B and C, 4.J2 expressing H681 showed significant enhanced infectivity in macrophages (P<0.05) compared to 4.J2 expressing Y681. In this assay, YU2 and JR-CSF Envs were used as positive and negative controls respectively [52]. Our data indicated that H681 modulated overall Env conformation that is more suitable for CD4 interaction and thus enhanced infectivity in low CD4 expressing cells.

The enhanced neutralization of H681 Envs over Y681 Envs by autologous and heterologous plasma pool led to the consideration of possibility that Y681H substitution might act on coreceptor binding site (CoRbs) and shift Env trimer to its open form mimicking CD4 bound state of gp160. Previous studies [53], [54] have showed that Env which exposes CoRbs in unbound state or the Envs which are independent of CD4 are extremely sensitive to antibodies. Thus, to test whether presence of H681 modulated exposure of CoRbs in Env, we examined the sensitivity of Envs expressing H681 and Y681 against 17b (that binds to CD4-induced epitope). Neutralization data (Figure S2) with 4.J2 and YU2 Envs showed enhanced sensitivity of H681 variants to 17b by >4 and >3 fold respectively (P<0.0001) suggesting that Y681H substitution essentially exposed CoRbs that is more reactive structure for binding with CD4 but more sensitive to neutralization.

### Y681H substitution displayed enhanced neutralization in unrelated Envs

In the present study, we examined whether introduction of H681 in place of much conserved Y681 in autologous and unrelated Envs (both clade C and non-clade C Envs) results in similar enhancement of Env sensitivity to neutralizing antibodies without compromising infectivity (Figure S3 and Table 1). This was done to evaluate if this substitution of highly conserved tyrosine to histidine at the 681 position (Y681H) results in global enhancement of neutralization sensitivity to Envs obtained from unrelated patients and that this effect was not patient specific. For this, we selected Envs that are refractory to neutralization to the reagents tested here. Thus, we substituted Y681H in two unrelated clade C patient-derived Envs (11-3.J3 and 2-3.J7) [46], Q259ENVd2.17 (clade A), YU2 (clade B) and CRF02_AG-235 (A/G recombinant) and tested Env-pseudoviruses carrying both H681 and Y681 in different clade backgrounds for their sensitivities to sCD4, b12, 4E10, 2F5 MAbs and plasma antibodies in addition to their infectivity in TZM-bl cells. The neutralization assays were carried in parallel with Env-pseudotyped viruses TND L669 and TND S669 [44] as well as Q461.e2, Q461.e2 (TA), Q461.e2 (IV) and Q461.e2 (TAIV) [43] (Table 1) to assess the degree of neutralization sensitivities of different Envs carrying Y681H in the context of these published data. As shown in Table 1, with Y681H substitution, all the Envs conferred increased sensitivities to sCD4 and 4E10 MAb (P = 0.0002 and 0.008 respectively). With respect to MAbs targeting MPER, we found that the sensitivity of 2-3.J7 and 11-3.J3 was increased by 4.6 and 3.2 fold respectively, whereas the increase in 4E10 sensitivity of YU2, Q259d2.17 and CRF02-AG235 Envs by over 30, 14 and 30 folds respectively was noticeable (P<0.05 for all comparisons). Interestingly, Y681H was found to increase 2F5 sensitivity of 11-3.J3 Env (clade C Env with rare presence of DKW motif in MPER) by >2-folds, this substitution conferred increased sensitivity of YU2 Env by 14-folds (P = 0.002); however presence of H681 in both Q259d2.17 and CRF02-AG235 Envs was not found to alter 2F5 sensitivities. All the Envs expressing H681,except 4.J2 and 11-3.J3, showed significantly greater sensitivity to b12 MAb than previously reported [43], [44]. The clade C Env, 2-3.J7, which we found sensitive to b12 MAb amongst several Indian clade C Envs obtained from recent infection showed a 8.8-fold increase in its sensitivity to b12 with the incorporation of H681. Similarly, H681 enhanced b12 sensitivity in Q259d217 (>4-folds), YU2 (>14 folds) and CRF02_AG-235 (>30-folds) (Table 1; P<0.05 for all comparisons). Besides, all the Envs bearing H681 showed enhanced sensitivity to sCD4 (between 4–125 folds). The remarkable modulation of CD4bs in all the Envs due to the presence of H681 resulting in significant enhancement of the sensitivity to b12 MAb and sCD4 was in contrast to any single amino acid change such as L669S [44], Q461.e2 (TA) and Q461.e2 (IV) [43] reported earlier, which resulted in marginal increment in CD4bs modulation.

### Effect of Y681H on gp120 shedding

**Figure 7:**
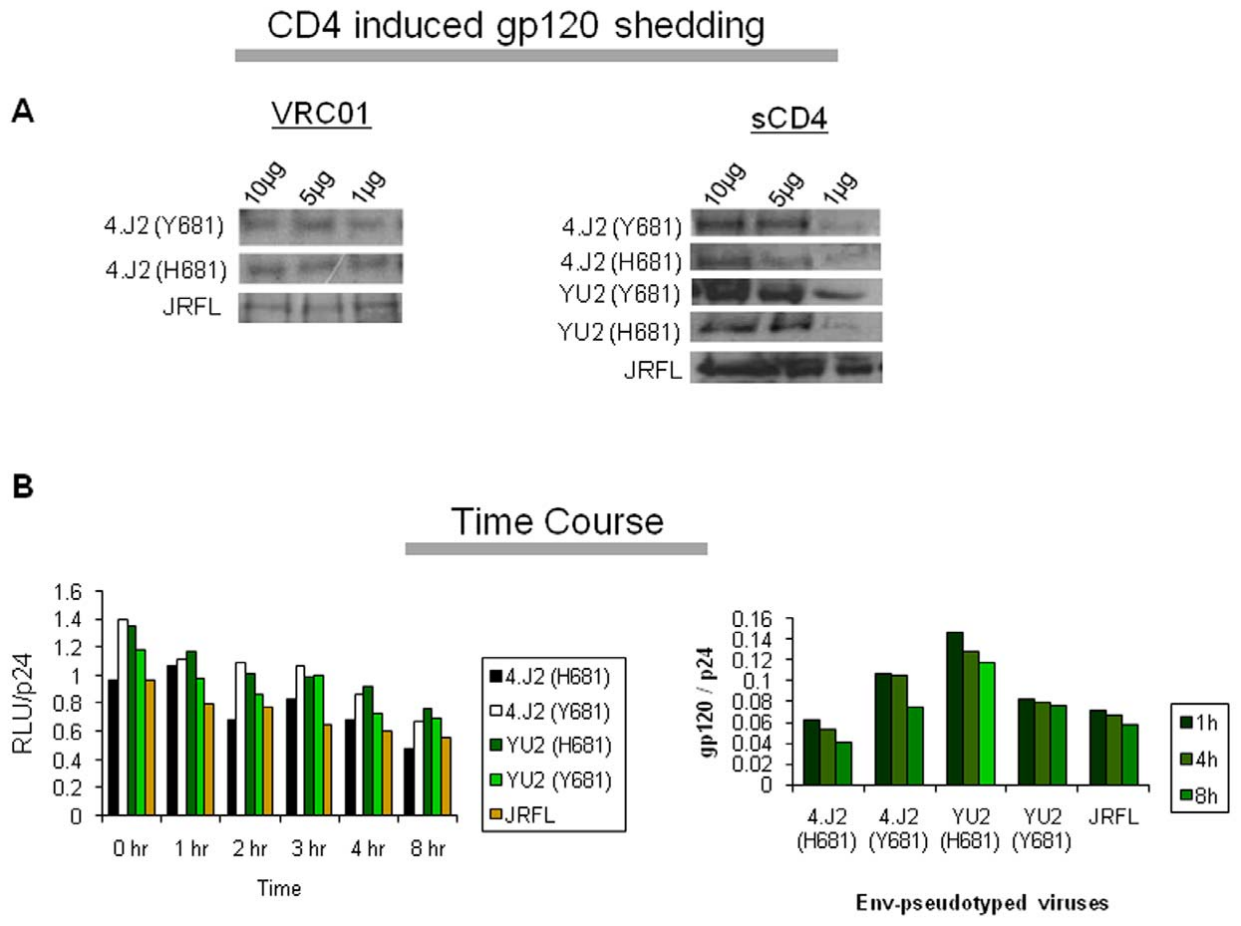
gp120 shedding assay. (A) The CD4-induced gp120 shedding assay was done by Western blot analysis of Envs obtained from supernatants of 293T cells transfected with the plasmid DNA encoding 4.J2 and YU2 Env variants as well as JRFL Env. HIV-1 gp120 shedding upon binding of sCD4 and VRC01 from the Env trimers on the surface of 293T cells was assessed by probing with the anti-gp120 human monoclonal antibodies (VRC01, 447-52D, 3074). (B). gp120 shedding of viral variants with respect to time. Env-pseudotyped viruses harvested from transfected 293T cells were incubated for indicated times at 37°C. The particle associated gp120 shedding was assessed by calculating the ratio of infectivity in TZM-bl cells and p24 (left panel) and ratio of band intensities of gp120 and p24 by densitometric analysis (right panel).

The possibility of Y681H substitution to reduce the subunit (gp120 and gp41) association on the viral membrane and spontaneous shedding of gp120 from the Env trimer was also considered and examined to explain its contribution in neutralization. To test this, we selected 4.J2, 4.J2 (H681Y), YU2 and YU2 (Y681H) and JRFL as control. We examined both CD4-induced shedding as well as spontaneous shedding with respect of time. Cell free viral variants were first quantitated by p24 antigen ELISA and normalized for quantitative assessment of dissociated gp120 in Western blot analysis. To test CD4-incuded gp120 shedding, 293T cells expressing Envs were treated with different concentrations of sCD4 and VRC01 MAb at 72 hours; cell supernatants were collected subsequently and run in 8% SDS-PAGE as described by Li *et al* [55]. As shown in Figure 7A, the shedding induced by sCD4 at various concentrations of sCD4 was found to be comparable between Envs expressing Y681 and H681. VRC01 MAb was taken as a negative control inducer of gp120 shedding as recently shown by Li *et al.* [55]. Furthermore, to evaluate time-dependent spontaneous gp120 shedding, we examined loss of infectivity and virus particle-associated gp120 and p24. Thus, cell supernatants containing Env-pseudotyped viruses were incubated at 37°C for different indicated times and while infectivity was assessed in TZM-bl cells, p24 was measured by ELISA. Virus containing supernatants collected at indicated times were pelleted in an ultracentrifuge and gp120 and p24 resolved in 10% SDS-PAGE under denaturing conditions. As shown in Figure 7B, the shedding of gp120 with respect to time was found to be comparable between Env-pseudotyped viruses expressing H681 and Y681. Thus, our data indicated that the Y681H substitution had no impact on the association of gp120 and gp41 subunits in the Env trimers and did not contribute to the increased neutralization sensitivity.

### Frequency of tyrosine to histidine substitution at position 681 in HIV database

We compared the Env sequences in HIV database (www.hiv.lanl.gov) to examine the frequency of substitution of conserved tyrosine at position 681. We found that >99% (6/2230 do not contain Y681) sequences in HIV database have tyrosine at position 681. Interestingly, H681 was found in two Envs including 4.J2 that were of Indian origin, while other three Envs was found to contain C681 or S681 (www.hiv.lanl.gov/content/sequence/EPILIGN/EPI.html). When compared between all the Env clones from this patient at different time points, except 4.J2, all of the Env variants contained Y681. Notably, we obtained 10 additional Env clones containing Y681 from the baseline visit from which 4.J2, 4.J22 and 4.J27 [46] Env clones were obtained; however all of them turned either non-functional or yielded unacceptable infectivity titers and therefore not included in any neutralization assays. Nonetheless, as shown by Kulkarni *et* al [56], one HIV-1 clade C Env clone (HIV-16845-2.22) obtained from an acutely infected Indian patient before seroconversion was found to contain histidine at position 681 (H681) but serine at position 668 (S668) and showed high sensitivity to both sCD4 and 4E10 MAb (IC_50_ of 1.0 and 0.1 µg/ml respectively). This suggested that H681, despite being rare in its frequency of occurrence, it was likely not introduced due to PCR error.

## Discussion

In the present study, we have identified a HIV-1 clade C Env variant with histidine in place of much conserved tyrosine at position 681 in gp41 MPER from a recently infected HIV-1 positive Indian patient that showed enhanced sensitivity to autologous and heterologous plasma antibodies. Subsequent investigation showed that 4.J2 wild type Env containing H681 exposed conserved neutralizing epitopes both in MPER and CD4bs in viral envelope and displayed proportionate enhancement of Env sensitivity to 4E10 and sCD4. Previous studies by Brunel *et al* [57] has shown that elongated MPER peptide up to the transmembrane domain (residue 683) increased 4E10 binding by fourfold as compared to shorter version (671–680). However, Y681A substitution in a MPER peptide did not improve the 4E10 binding indicating that Y681 or A681 did not contribute in antibody binding or this substitution did not impact the local structure relevant for neutralization. Later, similar finding was reported by Nelson *et al* [58] and Montero *et al* [59] about the binding of MPER directed antibodies to the series of alanine mutants of MPER peptides which suggest that the structure or conformation of MPER is not influenced by Y681A. Unlike Y681A substitution which reduces the MPER specific antibody binding (Montero *et al* [59]), Y681H substantially increased neutralization sensitivity to 4E10 in 4.J2. When H681 was transferred to unrelated Env clones with different genetic background and clades replacing the conserved Y681 the sensitivity of these Envs was found to enhance by several folds to MAbs targeting MPER and CD4bs, plasma antibodies as well as sCD4 which suggests that Y681H substitution influences the Env conformation so as to expose the NAb epitopes in both CD4bs in gp120 and MPER in gp41 that are otherwise hidden or insufficiently exposed to immune system. Previous studies that showed effect of gp41 mutations on both MPER and CDbs [43], [44] demonstrated greater effects on MPER epitopes than that a single amino acid change could impose on CD4bs. In our study, we found that Y681H enhanced sensitivity of Envs from distinct genetic background proportionately to antibodies targeting MPER and CD4bs as well as sCD4 and was higher in its magnitude particularly to b12 and sCD4 in contrast to those reported earlier [43], [44]. In addition to H681, we found N668S substitution in MPER also modulates Env sensitivity to sCD4; however was not found to affect Env sensitivity to plasma antibodies and 4E10. Back *et* al [60] demonstrated that although the same N668S increased Env sensitivity to sCD4, Env sensitivity to plasma antibodies was decreased. Our data also support this observation.

The presence of H681 in 4.J2 Env is unique compared to all the other Envs obtained either at the baseline or at subsequent time points from this particular patient [46], wherein all the Envs was found to bear much conserved Y681. This indicates that either H681 was a minor sporadically generated or it could be a transmitted virus exposing multiple epitopes as viruses in the early infection are not selected on the basis of antibody resistance but possibly on the basis of receptor and coreceptor affinity [61], [62]. A HIV-1 Env clone amplified from acutely infected Indian patient before seroconversion (HIV-16845-2.22) containing H681 has recently been reported [56] which also showed enhanced sensitivity to sCD4 and 4E10, indicating that 4.J2 Env containing H681 possibly represented the early stage strain though we did not find this Env to be less glycosylated which is usually a characteristic feature of early transmitted strains and therefore demand more focused study and analysis to come to such conclusion[63]. Although Shen *et al* [44] demonstrated the basis for increased exposure of neutralizing epitopes in MPER by a single L669S substitution; the basis for enhanced Env sensitivity to reagents interfering gp120-CD4 interaction such as sCD4 and IgG1b12 was less clear. In the present study, we found that H681 conferred exposure of CD4bs that resulted in increased infection in HeLa cells and macrophages both expressing low cell surface CD4. These observations indicated that the CD4bs on the HIV-1 envelope was modulated by a single amino acid change in a distant gp41 MPER thereby enhancing the capacity of 4.J2 expressing H681 to infect monocyte-derived macrophages. This observation was further strengthened when we found that the expression of H681 rendered Envs to over compete anti-CD4 MAb- QS1420 for relatively rapid engagement with cellular CD4 as opposed to Y681 suggesting that H681 enhances Env binding to CD4. The infectivity in low CD4 expressing cells such as macrophages often correlates with the sensitivity to reagents that block Env∶CD4 interaction suggesting a common determinants are involved to confer these phenotypes [52], [64]. Moreover, the enhanced neutralization sensitivity of H681 variants was not explained by the dissociation of gp120 from Env trimers as “shedding” effect because the gp120 dissociation was found similar for both Y681 and H681 variants of the corresponding Envs by either sCD4 or by spontaneous time dependent shedding. Taken together, the results suggest that the enhanced neutralization of Y681H mutants is primarily due to altered antigenic property of Env at the local site MPER as well as distal CD4 binding site.

Tyrosine (Y681) residue lies in LWYIK motif which is highly conserved and located immediately proximal to membrane spanning domain. Previous studies by Chen *et al* [65] have shown that deletion of this entire motif or YI (681–682) or IK (682–683) residues within this motif drastically reduced the membrane fusion and one-cycle viral replication but retained the Env incorporation onto the virus particles. It is possible that any change in this highly conserved motif likely would alter exposition of structural epitopes during fusion process. Previous studies have also described K683 is the N-terminal border of trans-membrane domain (TM) and LWYIK which contains Y681 is pre-TM helical segment [59], [65], [66], [67], [68], [69], [70]. Pre-TM region participates in formation and expansion of fusion pores [65], [68] and it is thought that this sequence specifically interact with cholesterol containing membranes as described by Saez-Cirion *et al* [71] and destabilize the membrane architecture around the anchoring transmembrane stalk of gp41. It is likely that Y681H substitution in part altered the property of Pre-TM and due to which the efficiency of formation and/or expansion of the fusion pore is reduced although end point infectivity of the viruses were not reduced (P = 0.324) (Figure S3). The structural changes in Pre-TM during the fusion process or the kinetics thereof might be altered due to Y681H substitution and likely modulated the 4E10 access and binding to the neutralization competent structure (NCS) in gp41 [59]. In addition to enhanced neutralization sensitivity to antibodies targeting MPER, H681 also increased Env sensitivity to enfuvirtide (T-20) which inhibits 6-helix bundle formation, supports the view that this substitution has affected virus fusion with the target cell membrane. This is in agreement with the observation by Shen *et al* [44], where they reasoned that increased sensitivity to T20 or MPER antibodies was due to prolonged exposure of MPER due to L669S mutation that they found. The formation of the pre-hairpin fusion intermediate following CD4 and coreceptor attachment of the viral envelope is characterized by the exposure of the N-terminal hydrophobic fusion domain and the C-terminal MPER of gp41, which are normally folded inside the gp41 trimers and not exposed to circulating antibodies [72]. These sites are transiently exposed to the antibodies during fusion. In the present study, we found significant increase in the T-20 sensitivity of Envs containing H681in different genetic backgrounds. Time course of 4E10 mediated neutralization of these Env versions along with L669S Env showed that structural intermediates recognizable by 4E10 existed until 6 min for H681 as opposed to 1 min for Y681, suggesting the enhanced sensitivity due to H681 was ascribed to differential exposure of MPER. Increased sensitivity to T-20 and neutralization of H681 Env pseudotyped virus (pre-adsorbed on cells) by 4E10 suggests enhanced exposure of MPER, however, this conclusion can better be drawn from the kinetics of antibody binding experiments that measure the rate of antibody binding or dissociation which is the major caveat of this study. Therefore, we conclude from the available body of data obtained in this study that single amino acid substitution Y681H leads to enhanced neutralization sensitivity to MPER directed antibodies as well as the antibodies to CD4bs. Studies by Sun *et al* [73] and Song *et al* [74] demonstrated that Y681 is buried into the membrane bilayers and N-terminal portion recognized by MPER specific MAbs has a kinked structure which maintains the hinge-related function of MPER. Also the data from Yue *et al* [67] and Montero *et al* [59] suggests that the transmembrane region starts even before than the 684 (which is the start of transmembrane domain according to the conventional classification) It is likely that Y681H substitution reduces the membrane immersion depth of this region pulling it outward or the movement of the local region modulating the exposure of epitopes for recognition by 2F5 and 4E10 MAbs during the structural transitions in fusion process.

Owing to the unavailability of crystal structure that include both the subunits of envelope protein together, it is difficult to know how a substitution in MPER region of gp41 affects the structural features at distant sites and modulate anti gp120 antibody binding and neutralization sensitivity. Although we do not know the antibody specificity in plasma pool that we tested in this study, it is likely that a portion of the antibodies was directed to gp120 protein [75]. Although Y681H substitution was found to expose CD4bs which harbors epitope for b12 MAb in addition to sCD4, Env clones such as 4.J2 and 11-3.J3 are refractory to b12 neutralization [46], indicating that b12 epitope is too mutated to be sampled by antibody in these envelopes [76].While the conformational changes plausibly explain the enhanced antibody binding and Env sensitivity to the plasma antibodies, b12 MAb and sCD4, it underscores the need for the detailed structural analyses to precisely dissect the basis for such phenotype. Identification of novel motifs in Env capable of enhancing neutralization potential of HIV-1 would contribute valuable information to the existing neutralization signatures [4] that might help in designing strategies for developing vaccine capable of inducing broadly neutralizing antibodies.

## Materials and Methods

### Ethics statement

Ethical clearance of this study was specifically obtained from the National AIDS Research Institute (NARI) Institutional Review Board (IRB) prior to initiation of the study. Written informed consent was taken from participants involved in this study. Autologous plasma and peripheral blood mononuclear cells (PBMC) of IVC4 patient from which *env* clones were obtained was reported previously [46]. Heterologous plasma samples were obtained from patients during their visit at NARI clinic for routine testing. PBMCs from HIV negative healthy donors for preparation of monocytes were obtained following ethical clearance by NARI IRB.

### Patient samples, Env clones and cells

Clade C Env clones (obtained from infected Indian patient) used in this study were described recently [46]. Ethical clearance of this study was specifically obtained from the National AIDS Research Institute (NARI) Institutional Review Board (IRB) prior to initiation of the study. Written informed consent was taken from participants involved in this study. Heterologous plasma samples were obtained from chronically infected patients with CD4 counts above 450 cells/mm^3^ from the National AIDS Research Institute clinic. Patient samples were obtained with prior approval of institutional ethics review board (IRB). pSVIIIenv-YU2 and pSVIIIenv JRCSF plasmids were kindly provided by Dr Paul Clapham, UMASS Medical School, Worcester, Massachusetts, USA. Engineered HeLa cells (RC49 clone) were kindly provided by Dr David Kabat, University of Oregon, Portland, USA. L669 and S669 plasmids [44] were kindly provided by Dr Georgia Tomaras, Duke University, Durham while Q461.e2, Q461.e2 (TA), Q461.e2 (IV and Q461.e2 (TAIV) [43] were kindly provided by Dr Julie Overbaugh, University of Washington, Seattle. Q259ENVd2.17, CRF02AG235 Env clones and pNL-AD8 [77]were obtained through NIH AIDS Research Reagents and Reference Program, Division of AIDS, NIAID, NIH from Dr Julie Overbaugh and Drs. Ellenberger, D., Li, B., Callahan, M., and Butera, S respectively.

### Construction of envelope chimera, mutagenesis and cloning

Env clones were amplified from patient's peripheral blood mononuclear cell (PBMC) without culture as described before [46]. Chimeric envelopes were made by swapping V1V2 by using HindIII and BbvCI and gp41 fragments using BbvCI and NotI between sensitive and resistant Envs as shown in Figure 1. Single point mutations were introduced by site-directed mutagenesis using the In-Fusion Dry-Down PCR Cloning kit (Clontech Inc.) following manufacturer's protocol. Briefly, specific substitutions were introduced by PCR using high fidelity proof reading polymerase (Platinum Taq, Invitrogen Inc.); subsequently, single stranded overhangs were created in amplicons by exonuclease activity of pox DNA polymerase provided in the kit. These fragments were then annealed with each other at 37°C and transformed to *E.coli* Top10 cells (Invitrogen Inc.). Chimeric and mutant version of the Envs was confirmed by sequencing.

### Pseudovirion preparation and measurement of virus titer

Pseudotyped viruses carrying patient envelope genes were produced by cotransfection of Env^+^ pcDNA 3.1/V5-His-TOPO with Env-defective HIV-1 backbone vector (pSG3ΔEnv) [16] into 293T cells (obtained from American Type Culture Collection; ATCC) during log growth phase in 6-well tissue culture trays (Corning Inc) using calcium phosphate (Promega Inc) following manufacturer's protocol. Cell supernatants carrying progeny pseudotyped viruses were harvested at 48 hours post-transfection, and stored at −80°C until further usage. The infectivity assays were done in TZM-bl cells in 96-well microtiter plate and infectivity titers determined by measuring the luciferase activity respectively as described elsewhere [78].

### Preparation of monocyte derived macrophages

Monocyte-derived macrophages were prepared from peripheral blood mononuclear cells (PBMCs) from healthy donors followed by ethical clearance by National AIDS Research Institute Institutional Review Board. Briefly, PBMCs were separated by using Ficoll gradient and were carefully collected from the buffy coat ring and were washed in RPMI-1640 to remove impurities. Monocytes were prepared from PBMCs using Dynabeads MyPure Monocyte Kit 2 (Invitrogen Inc.) following manufacturer's protocol. Monocytes were plated in T75 tissue culture flask (Corning Inc.) in presence of Dulbecco's modified Eagle's medium (DMEM) supplemented with 10% fetal bovine sera (FBS) and 100 ng/ml of macrophage-colony stimulating factor (M-CSF) (Sigma Inc.) and incubated at 37°C in a CO_2_ incubator. The non-adherent cells were removed the following day and adhered cells were further incubated for 5–7 days for differentiation. Macrophages were gently removed with the help of Versene (Invitrogen Inc.), and scrapping with cell scrapper, washed with DMEM and 2×10^4^ cells were plated in 96-well tissue culture tray and further incubated for overnight at 37 C in a CO_2_ incubator. Macrophages were infected with Env-pseudotyped viruses by spinoculation as described previously [79], [80]. In addition, commercially available monocytes (obtained commercially from Lonza Inc.) were also used to prepare macrophages following manufacturer's protocol. In all cases, monocytes were allowed to adhere in plastic plates and were incubated with M-CSF as described above for differentiation to macrophages.

### Neutralization assays

Neutralization assays were done by measuring reduction in luciferase activity in a single round infection of TZM-bl cells with Env-pseudotyped viruses as described earlier [46]. Antibody titers were calculated as the inhibitory concentration (IC_50_) for MAbs and reciprocal dilution (ID_50_) for plasma causing 50% reduction of relative light units measured in a luminometer (Perkin Elmer Inc.)

### Time Course of 4E10 Neutralization Assay

The time course of neutralization of Envs by 4E10 MAb was determined in a synchronized post-attachment HIV-1 pseudotyped virus neutralization assay as described earlier [44]. Briefly, TZM-bl cells (1×10^4^/well) were plated and allowed to adhere overnight. The plate was then cooled on ice for 15 minutes followed by addition of cold Env pseudotyped viruses. Virus was allowed to adhere on cells for 2 hours on ice. To remove unbound viruses, plates were washed with fresh, cold medium. Warm medium (150 µl/well) was added to each well followed by 100 µl of inhibitory concentrations of 4E10 MAb (10 µg/ml) at different time intervals (0, 5, 10, 15, 20, 25, 30 and 60 min). Infectivity was measured by relative light units (RLUs) as described above at 48 hours.

### Env-anti CD4 competition assay

The competition for binding of anti-CD4 MAb and Env-pseudotyped viruses to cellular CD4 was carried out in TZM-bl cells. For this, mouse monoclonal antibody, QS4120 (Calbiochem, Inc.), which binds to domain 1 of CD4 and blocks binding of gp120 to CD4 was used. Briefly, QS4120 MAb was serially diluted (2-fold) in 50 µl of growth medium in a 96-well tissue culture tray (Corning Inc.). 50 µl pseudotyped virus (containing equal infectious titer) of each Env was then mixed with serially diluted antibody.100 µl TZM-bl cells were subsequently added onto this mixture and further incubated for 3 hours in a CO_2_ incubator at 37°C. Residual viruses were removed gently after 3 hours and replenished with fresh growth media. The plate was further incubated for additional 2 days in a CO_2_ incubator at 37°C. Virus infectivity was measured as a function of RLU in a luminometer.

### gp120 shedding assay

#### CD4-induced gp120 shedding

The CD4-induced gp120 shedding was examined as described by Li *et al.* [55]. Briefly, 293T cells were co-transfected with Env plasmids and pSG3Δenv. Forty-eight hours post transfection, approximately 1×10^7^ cells were collected and washed with phosphate buffered saline; PBS (pH 7.4). Two-hundred-micro liter aliquots of the cell suspension were taken into micro centrifuge tubes for sCD4 or VRC01 at a given concentration. The cells in microfuge tubes were spun at 350× g for 5 minutes and a total of 160 µl of the supernatant was removed and discarded. 10 µl of the ligand solutions were then added to the cells to make final concentrations as 10 µg/ml, 5 µg/ml and 1 µg/ml. Cells were mixed and incubated at 37°C for 2 hours with gentle mixing every 20 min. The cells were then subjected to centrifugation, and the supernatants were transferred to fresh tubes. A total of 25 µl of each supernatant was resolved in 8% SDS-PAGE. The proteins were transferred to PVDF membranes for Western blotting. To identify gp120, 1∶1 mixture of VRC01, anti V3 MAbs 447-52D (in case of YU2 and JRFL) and 1∶1 mix of VRC01 and anti-V3 MAb 3074 [81] (in case of 4.J2) was used as a primary antibody, followed by a horseradish peroxidase (HRP)-conjugated anti-human secondary antibody (Thermo Scientific, Inc.) and blots were developed with luminol substrate (Super Signal West Dura kit; Thermo Scientific Inc.).

#### Time-dependent gp120 shedding

To assess spontaneous gp120 shedding in a time-dependent manner, 293T cells were co-transfected with Env plasmids as described above and virus contining supernatants collected after two days. The culture supernatants containing Env pseudotyped viruses were filtered through a 0.45 µm filter to remove any cell debris and subsequently incubated at 37°C for various times (0, 1, 2, 3, 4, and 8 h). At each time point, a sample of each virus was collected and frozen at −80°C. The virus stocks were later thawed together and tested for single-cycle infectivity in TZM-bl cells and p24 by ELISA. Virus supernatants incubated for 1, 4 and 8 hours were further pelleted by ultracentrifugation for 1 hour in presence of 20% sucrose containing 10 mM Tris-Cl (pH 7.4), 100 mM NaCl,1 mM EDTA at 35,000× g at 4°C [82]. Virus pellets were dissolved in 30 µl PBS and resolved in 10% SDS-PAGE gel. gp120 and p24 were developed by Western blotting using HIV+ patient sera pool (1∶100). The relative levels of particle-associated gp120 were calculated as the ratio of the infectivity in TZM-bl cells and p24 concentrations and ratio between band intensities between gp120 and p24 (by densitometry analysis).

### Cell-Based Enzyme-Linked Immunosorbant Assay (CELISA)

The binding of MAbs to HIV-1 Env trimers expressed on cells was measured using a cell-based ELISA system, as described by Haim *et al* [83] and Wilen *et al* [84]. Briefly, 293T cells were seeded in 96-well plates (3×10^4^ cells/well) and transfected the following day with 100 ng of Env expressing plasmid and 200 ng of pSG3ΔEnv backbone plasmid per well using lipofectamine 2000 (Invitrogen Inc). Three days later, the growth medium was discarded and cells were gently washed with PBS. After washing cells were incubated with IgG1b12 or CD4-Ig in blocking buffer (35 mg/ml BSA, 10 mg/ml non-fat dry milk, 1.8 mM CaCl2, 1 mM MgCl2,25 mM Tris, pH 7.5 and 140 mM NaCl) for one hour at room temperature. Cells were then washed four times with blocking buffer and four times with washing buffer (140 mM NaCl, 1.8 mM CaCl2, 1 mM MgCl2 and 20 mM Tris, pH 7.5). A horseradish peroxidase (HRP)-conjugated antibody specific for the Fc region of human IgG was then incubated with the samples for 45 minutes at room temperature. Cells were washed five times with blocking buffer and five times with washing buffer. HRP enzyme activity was determined after the addition of 40 µl of 1∶1 mix of Western lightning oxidizing and luminol reagents (Thermo Scientific Inc.). Binding of IgG1b12 or CD4-Ig was measured as a function of light emission (relative luminescence units) in a luminometer (Victor 4, Perkin Elmer Inc).

### Statistical analysis

Statistical analyses were performed using Graph Pad prism (version 5.1). To calculate the statistical significance (P values) between the ligand binding to H681 and Y681 variants of Envs in CELISA assay, we took more than five values (RLU) for each Env and significance of difference between the medians was calculated by paired t test. The multiple values for each Env were obtained from independent assays done in triplicates. P value less than 0.05 was considered significant. Similar tests were also applied to calculate P values for the difference in macrophage infectivity of H681 and Y681 Env variants. For assessing the differences in the sensitivities of Env-pseudotyped viruses to T20 and 17b, we used IC50 values for each Env pair (H681 and Y681) obtained from at least three independent assays and applied paired t test to examine whether mean IC50 values were significant. Spearman's rank correlation was applied to evaluate the correlation between the Env sensitivity to 4E10 and their infectivity to TZM-bl cells. For this, average IC50 values of 4E10 and average percent infectivity of H681 variants to Y681 variants of Envs were used.

## Supporting Information

Figure S1 **Effect of Y681H substitution on degree of inhibition of Env-pseudotyped viruses by T20.** The percent reduction in infectivity of each virus indicated on Y-axis at various dilutions of T20 indicated on X-axis in TZM-bl cells were assessed by measuring the reduction in relative luminescence units (RLU) in a luminometer. Experiments were done in duplicates and repeated at least three times. Note that Envs expressing H681 showed increased inhibition by T20 than the Y681 version of respective Env (P = 0.04).
(TIF)

Figure S2 **Effect of Y681H substitution on exposure of CD4-induced epitopes.** The effect of Y681H on relative exposure of coreceptor binding sites was assessed by examining Env sensitivity to coreceptor mimetic 17b MAb. The percent inhibition in infectivity of Env-pseudotyped viruses in TZM-bl cells indicated on Y-axis was assessed by measuring the reduction in relative luminescence units (RLU) at various concentration of antibody indicated on X-axis. Experiments were done in duplicates and repeated at least three times. Note that Env expressing H681 are more sensitive than Y681 versions suggesting shift towards CD4-bound-like structure with the coreceptor binding site more exposed.
(TIF)

Figure S3 **Effect of Y681H on infectivity of Env-pseudotyped viruses in TZM-bl cells.** Effect of Y681H substitution on infectivity of Env-pseudotyped viruses in different genetic backgrounds with equal virus particles (p24) was assessed in TZM-bl cells. TND L669S Env that was shown by Shen *et al* [44] to significantly enhance Env sensitivity to neutralizing antibodies was used as control. Percent infection of Y681H mutants or H681Y mutant (in case of 4.J2) relative to their wild type counterparts is represented on Y-axis.
(TIF)
