# Supplementary figures and images for: Association of Enhanced HIV-1 Neutralization by a Single Y681H Substitution in gp41 with Increased gp120-CD4 Interaction and Macrophage Infectivity
Source: PLoS One. 2012 May 14;7(5):e37157. doi: 10.1371/journal.pone.0037157 (PMC3351407; doi:10.1371/journal.pone.0037157)

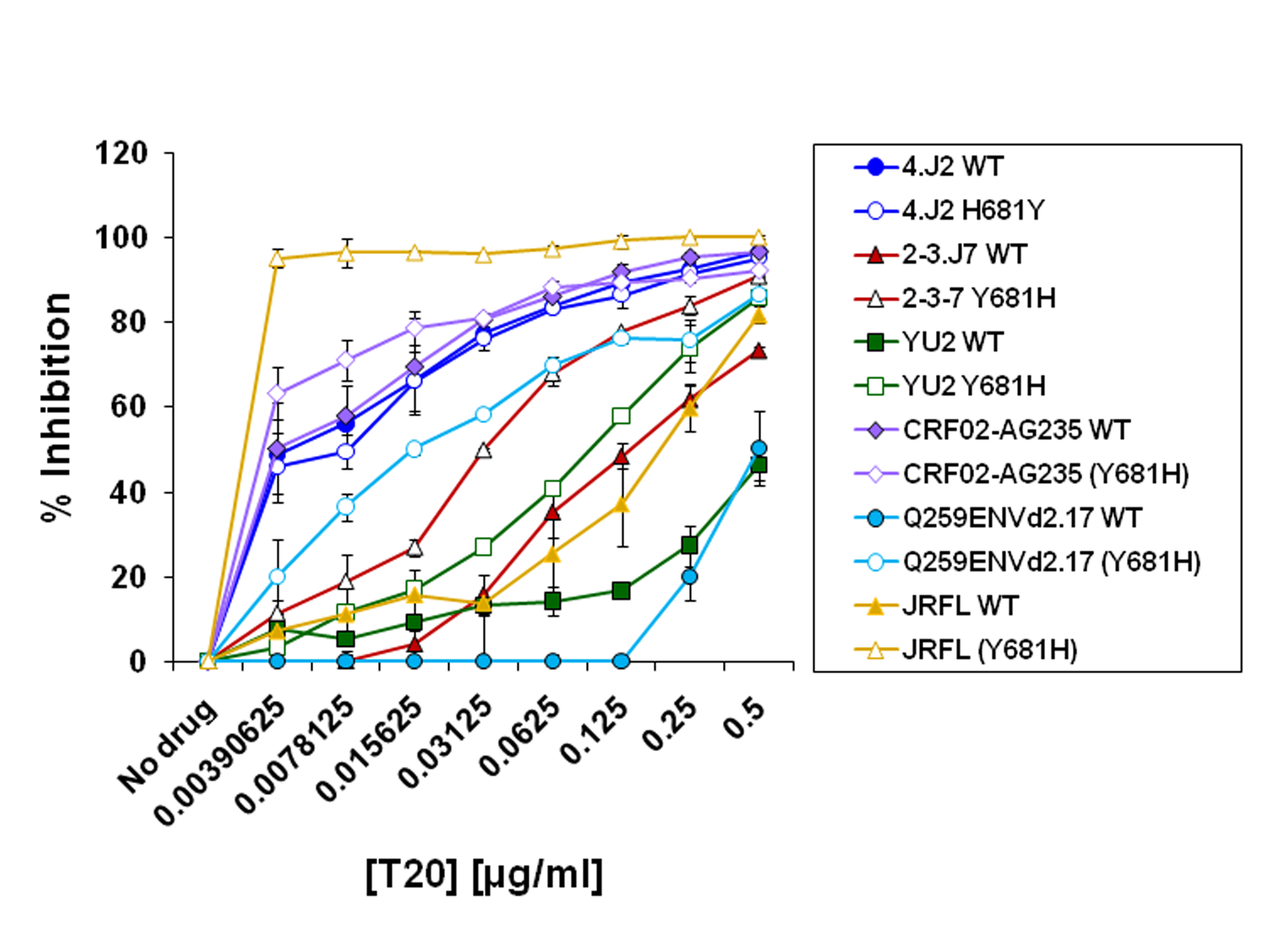

Supplement: Figure S1 — Effect of Y681H substitution on degree of inhibition of Env-pseudotyped viruses by T20. The percent reduction in infectivity of each virus indicated on Y-axis at various dilutions of T20 indicated on X-axis in TZM-bl cells were assessed by measuring the reduction in relative luminescence units (RLU) in a luminometer. Experiments were done in duplicates and repeated at least three times. Note that Envs expressing H681 showed increased inhibition by T20 than the Y681 version of respective Env (P = 0.04). (TIF) [file pone.0037157.s001.tif]

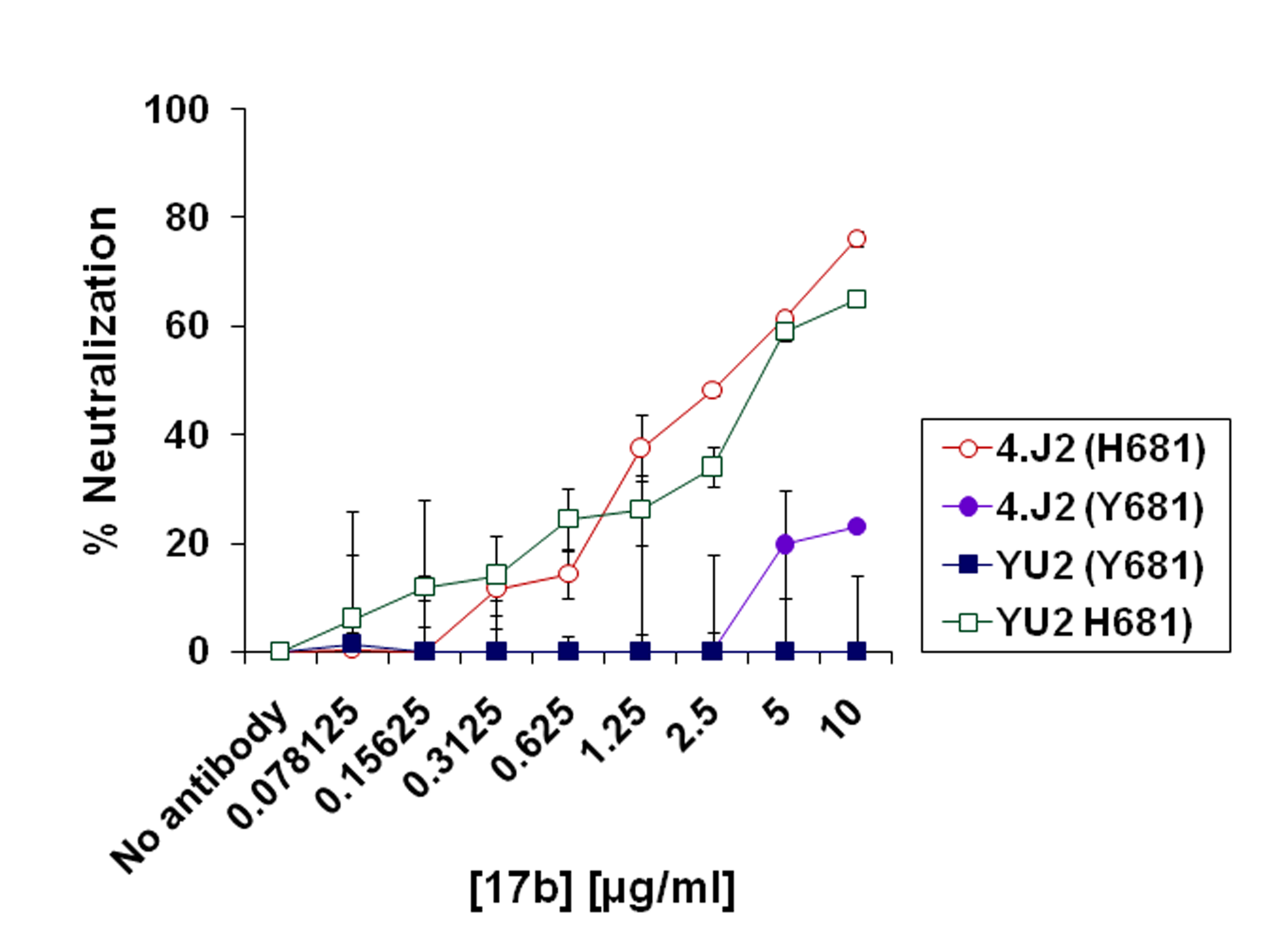

Supplement: Figure S2 — Effect of Y681H substitution on exposure of CD4-induced epitopes. The effect of Y681H on relative exposure of coreceptor binding sites was assessed by examining Env sensitivity to coreceptor mimetic 17b MAb. The percent inhibition in infectivity of Env-pseudotyped viruses in TZM-bl cells indicated on Y-axis was assessed by measuring the reduction in relative luminescence units (RLU) at various concentration of antibody indicated on X-axis. Experiments were done in duplicates and repeated at least three times. Note that Env expressing H681 are more sensitive than Y681 versions suggesting shift towards CD4-bound-like structure with the coreceptor binding site more exposed. (TIF) [file pone.0037157.s002.tif]

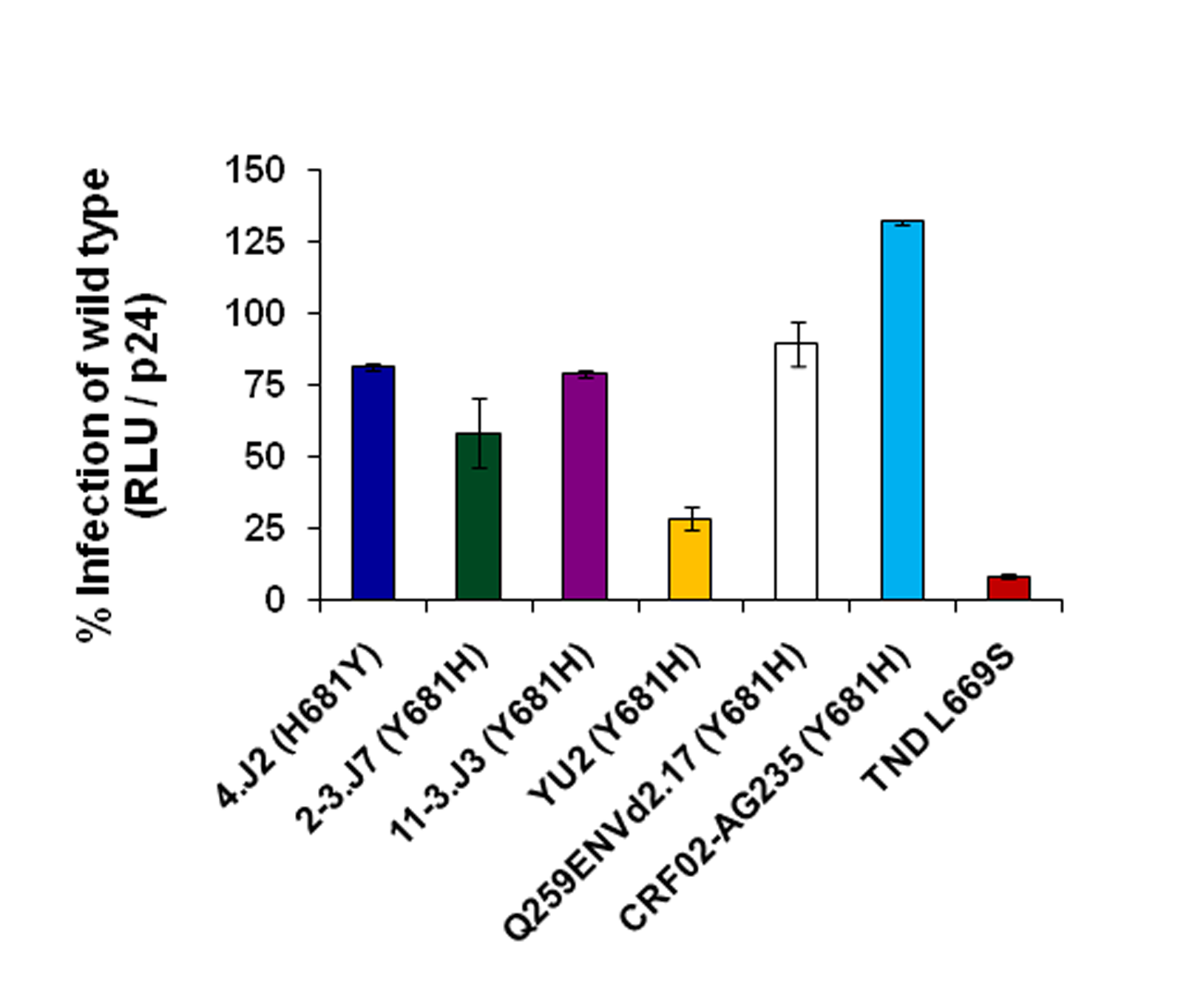

Supplement: Figure S3 — Effect of Y681H on infectivity of Env-pseudotyped viruses in TZM-bl cells. Effect of Y681H substitution on infectivity of Env-pseudotyped viruses in different genetic backgrounds with equal virus particles (p24) was assessed in TZM-bl cells. TND L669S Env that was shown by Shen et al [44] to significantly enhance Env sensitivity to neutralizing antibodies was used as control. Percent infection of Y681H mutants or H681Y mutant (in case of 4.J2) relative to their wild type counterparts is represented on Y-axis. (TIF) [file pone.0037157.s003.tif]
